# Field-recorded data on habitat, density, growth and movement of *Nephrops norvegicus*

**DOI:** 10.1038/s41597-019-0013-x

**Published:** 2019-03-26

**Authors:** Anne Marie Power, Julian Merder, Patricia Browne, Jan A. Freund, Liam Fullbrook, Conor Graham, Robert J. Kennedy, Jack P. J. O’Carroll, Alina M. Wieczorek, Mark P. Johnson

**Affiliations:** 1 Ryan Institute, National University of Ireland Galway, University Road, Galway, Ireland; 2 Institute for Chemistry and Biology of the Marine Environment, University of Oldenburg, Oldenburg, Lower Saxony Germany; 3 Marine & Freshwater Research Centre, Galway-Mayo Institute of Technology, Dublin Road, Galway, Ireland

## Abstract

The availability of growth data in *N. norvegicus* is important for management purposes due to a lack of aging criteria and the commercial importance of fisheries in this species. Growth varies as a function of stock density, hence comparisons of growth rates between stocks at known density is particularly valuable. Growth is also related to starting size in males, making raw data on size-specific growth rates more valuable. Internally injected passive tags allowed us to track the growth of male and female individuals over one or two years. The spatial position of tagged recaptures was recorded to measure site fidelity of tagged releases. A total of 3300 pots were fished and their spatial positions were recorded to enable Catch Per Unit Effort calculations. Similarly, spatially geo-referenced v-notching and notched recovery enables spatially gridded densities to be calculated. Finally, acoustic mapping was carried out both on and off the fishing ground and was ground-truthed with sedimentology from grabs at 22 stations. These data are useful for fisheries and macroecological studies.

## Abstract (metadatasummary)

**Table:** 

Design Type(s)	observation design • organism development design • longitudinal study design
Measurement Type(s)	growth • habitat
Technology Type(s)	observational method
Factor Type(s)	geographic location • biological sex • notched
Sample Characteristic(s)	Nephrops norvegicus • Ireland • ocean biome

Machine-accessible metadata file describing the reported data (ISA-Tab format)

## Background & Summary

*Nephrops norvegicus* is a small lobster in North-East Atlantic Europe and the Mediterranean where it occupies burrows in muddy substrates^1^. It is commercially important in Europe, being fished in at least 34 Functional Management Units and comprising thousands of tonnes of landings in the United Kingdom, Ireland, and to a lesser extent, in France, Denmark, Iceland and Sweden^2^.

Fisheries management in *N. norvegicus* is compromised by a lack of reliable aging methods, which in turn affects estimates of growth and recruitment, including how quickly new individuals reach minimum landing size. Indirect methods to estimate growth include length-cohort analysis, which follows size cohorts through time to see how fast a modal size group progresses^3–7^, however this has been hampered by the relatively ‘flat’ size structure characteristic of many crustacean species^8^. Although proposed aging methods *via* ring structure analysis of the eyestalk or gastric mill (within the stomach) seemed initially promising, these structures are now thought to be lost on moulting^9^. Hence growth data in this commercially-important group are indirect or absent through most of the range. Neither is tagging straightforward because crustaceans moult and need to be tagged with internal tags. This technology was used in the present study and supplements a limited number of tagging studies in the Skagerrak^3^ and the west coast of Scotland^4,10^, with poor returns of tagged individuals having hampered this activity elsewhere^11^.

Much of the habitat requirements and drivers of density in *N. norvegicus* are derived from fisheries-associated data^12–14^, but pot fisheries in inshore areas are less studied and the distribution is more obscure. For example, inshore populations are known to inhabit both shallow and deeper water habitats (such as fjords^15^) where they may modify their emergence behavior according to ambient light^16,17^. Growth and density are thought to interact in this species due to various density-dependent signals^1,4,13,18^. Mean body size and L_max_ (theoretical size maximum) negatively correlate with density^1^. A mechanism has been shown for reduced body size at high densities in males, which has been linked to suppressed growth of inferior males in high density patches, although density-dependent mortality could not be detected in adult *N. norvegicus*^19^. Data to enable density calculations are provided in the present study.

A tagging programme was carried out in inshore grounds to provide information on the growth of individuals after one year (n = 205) or two years at liberty (n = 36), with approximately equal numbers of recaptured males (n = 108) and females (n = 132) (sex was not recorded for one individual). This enables growth to be compared between the study site in the west of Ireland and other parts of the biogeographic range, or allows data to be filtered according to particular starting sizes (starting size significantly affects growth in males^20^). Distance travelled between initial release point and final recapture point is given per individual. This provides some information about the site fidelity of lobsters e.g. for stock enhancement purposes. Size distribution of catch (tagged, v-notched and untagged) was recorded on 4742 occasions. These data are not all unique since some individuals will have been recaptured numerous times (as shown by re-capture of v-notched individuals). When v-notched individuals (n = 1093) were recaptured (n = 531), their recapture status was noted to enable density calculation using mark-recapture methods. Spatial information is provided for the pot in which every individual was captured as well as pots with no catch to enable estimation of density indices such as Catch Per Unit Effort to be calculated in raster format. Acoustic bottom type classifications are provided as these may be informative about *N. norvegicus* catch rates. Acoustic surveys were complemented by sediment grab samples to match acoustically derived sediment classes to standard sediment categories.

## Methods

### Experimental design

**Fig. 1:**
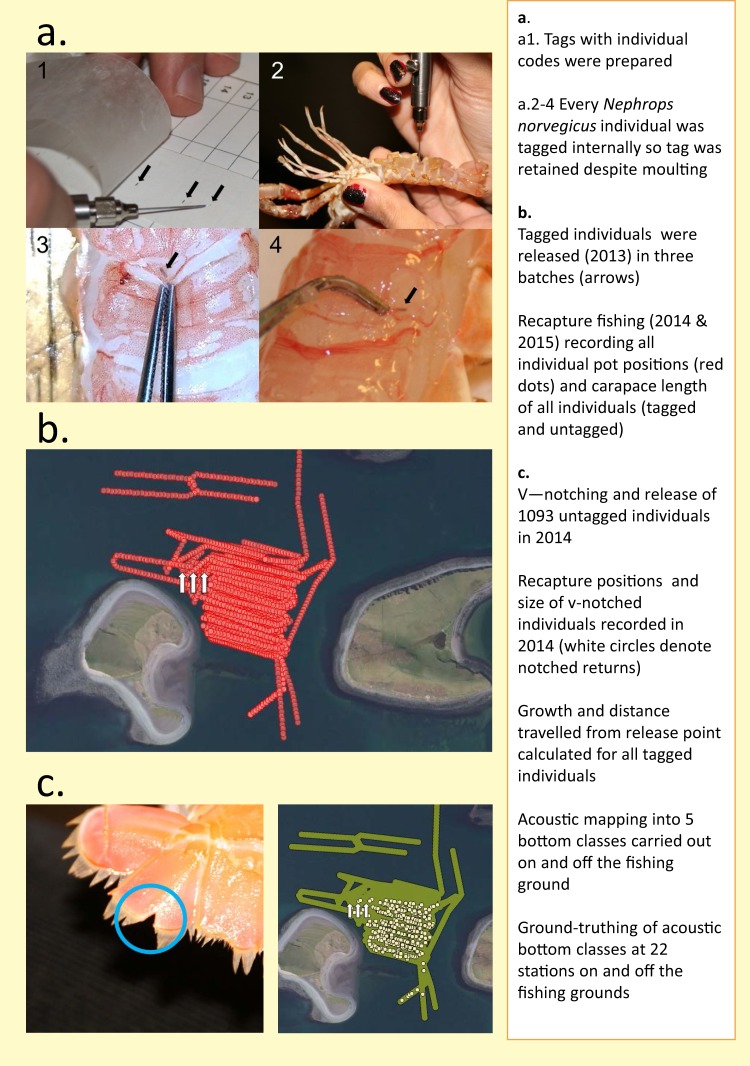
Experimental workflow used to generate and analyse the data descriptor. (**a**) Tag preparation and injection of tags internally in abdomen of *N. norvegicus*. (**b**) Release points of three batches of tagged individuals (white arrows) and recapture fishing pots (red dots). (**c**) (left) v-notching of telson (tailfan) of an individual and (right) fished area marked in green with positions of v-notched recaptures (white dots). The text panel on the right hand side describes the workflow.

Figure 1 shows a schematic of the experiment. We adopted the following methodology to collect the data of the three datasets^21^:

1. We released 1177 tagged *N. norvegicus* (males and females) at the fishing ground on 3 dates in 2013. 205 of these individuals were recaptured in 2014 and a further 36 individuals were recaptured in 2015 (see *N. norvegicus* sampling).
2. The starting size and size at recapture of tagged individuals was recorded, which allowed growth to be calculated. Date at recapture was also recorded to supplement growth data and provide a growth rate over time (i.e. number of days at liberty).
3. V-notching of 1093 untagged individuals was carried out in 2014 to enable population density estimates from mark-recapture methods^22,23^.
4. The spatial position of all 4742 individuals captured (tagged, notched and untagged) in pots was recorded by taking note of the position of the pot they were captured in.
5. The position of all 3300 pots including pots without catch was recorded to enable calculation of Catch Per Unit Effort (CPUE).
6. The size of all individuals in point 4., above, was also recorded with the exception of three individuals for whom size data were lost, giving a total of n = 4739 size data.
7. For tagged individuals, the distance moved between the release and recapture point was calculated per individual.
8. Notes were taken of whether females were ‘berried’ (bearing embryos), and whether individuals were recently moulted, as indicated by an exceptionally clean or soft shell.
9. To understand how density of *N. norvegicus* related to habitat variables, 4894 acoustic measurements were taken to provide 5 categories of bottom type (see ‘Acoustic data’) and these were ground-truthed using sediment size analysis at 22 stations (see ‘Sedimentology’). Acoustic mapping took place both in areas on the fishing grounds and outside the fishing grounds. Sediment size sampling was adjacent to the fishing grounds but not over the fished area. This exercise was intended to place acoustic signals into context rather than to directly link sediments to biological data.

### *N. norvegicus* sampling

Sampling was carried out via baited ‘pots’ (or ‘creels’). To prepare for tagging, experimental *N. norvegicus* were captured in a bay close-by the experimental area and held at an aquaculture facility until they could be tagged with Coded Wire Tags (CWTs - Northwest Marine Technology Inc.). Tagged individuals were released in a ‘controlled’ manner in 3 batches on separate dates as follows: 5 June, 19 June and 17 July 2013. Release dates were recorded per tagged individual to enable time at liberty and growth per unit time (in days) to be calculated. Coordinates for the three release positions were according to Irish Transverse Mercator projection and translated by a fixed offset (to protect anonymity of fishing grounds), as follows: release point 1 (X 650278, Y 656225), point 2 (X 650241, Y 656226) and point 3 (X 650314, Y 656224). The middle (point 2) of these three positions was used to calculate distance moved between the release site and the site of recapture for every tagged individual. After releasing tagged individuals, the fishing grounds were left ‘fallow’ (no fishing) until recapture fishing which took place in April to September 2014. Recapture fishing was carried out on 14 dates in 2014 when salted herring was used as bait in a series of 48 pots on a ‘string’, each pot being 10 m apart from its neighbour. All individuals captured were scanned and those with a tag were measured (Carapace Length - CL) and retained for return to the laboratory and further analysis. All individuals that did not bear a tag were measured, v-notched on the telson and returned to the seafloor, subject to a minimum size limit of 15 mm CL for v-notching females or 25 mm CL in males (although very few individuals of such small sizes were captured). Presence of a v-notch and size of individual was noted during subsequent fishing. In June and July of 2015 when tagged *N. norvegicus* had been at liberty for approximately two years, further recapture fishing was carried out and tagged individuals were again measured and returned to the laboratory. Sampling in 2015 did not record the untagged or notched individuals. At the laboratory, tags were dissected and read under a microscope (25X), individuals were identified and their starting sizes/final sizes compared to enable growth to be calculated. (See^20^ for further experimental details). A total of 3300 pots were fished during recapture fishing (to retrieve tagged/notched as well as ‘free catch’ individuals). As mentioned above, pots were attached to ‘strings’ with each string containing 48 pots fastened every 10 m apart from each other. A sample of up to five pots on each string contained GPS-coordinate information, always including the first and last pot in each case. Coordinates for all other pots on the specific string were interpolated. For each string we line-dashed the complete string length based on the sample of pot coordinates of the string using the QGIS^24^ addon ‘Points2one’ (https://launchpad.net/points2one). We then used the ‘QChainage’ addon (https://github.com/mach0/qchainage) to interpolate pot coordinates for the leftover pots on the string, based on the fact that they lie 10 m apart from each other. Comparison of the release/recapture locations allowed the distance travelled from the release point at recapture to be calculated.

### Acoustic data

Acoustic survey was carried out using the Biosonics MX echosounder which uses a single beam 200 kHz transducer with an 8.5 degree beam angle, at a fixed ping rate of 0.5 pings per second and fixed pulse duration of 0.4 ms. The MX has a depth range of 0–100 m and an integrated Differential Global Positioning System (DGPS) for georeferencing with a DGPS update rate of 1 second. The transducer is connected to a laptop in the field computer, running Visual Acquisition, a free software that configures and controls the echosounder, visualises the data acquired by the echosounder and related sensors and logs the data in a format that can be played back in a variety of post processing and data analysis packages. Further software from Biosonics allows identification of the sea floor and statistical processing using PCA (Principal Component Analysis) to interpret the signal and identify the composition of the seabed^25^. The classification is ‘unsupervised’: a number of sediment types must be specified at the start of the analysis. Once the number of sediment types is fixed, the software seeks distinct categories (clusters) using a Fuzzy Centroid Mean (FCM) algorithm. A total of 48 variables are extracted from the signal and summarized into the limited number of groups specified by the user. Results are available in the form of a 3-D scatter graph of similarity among pings and in a map view showing the distribution of the classified types^25^.

**Fig. 2:**
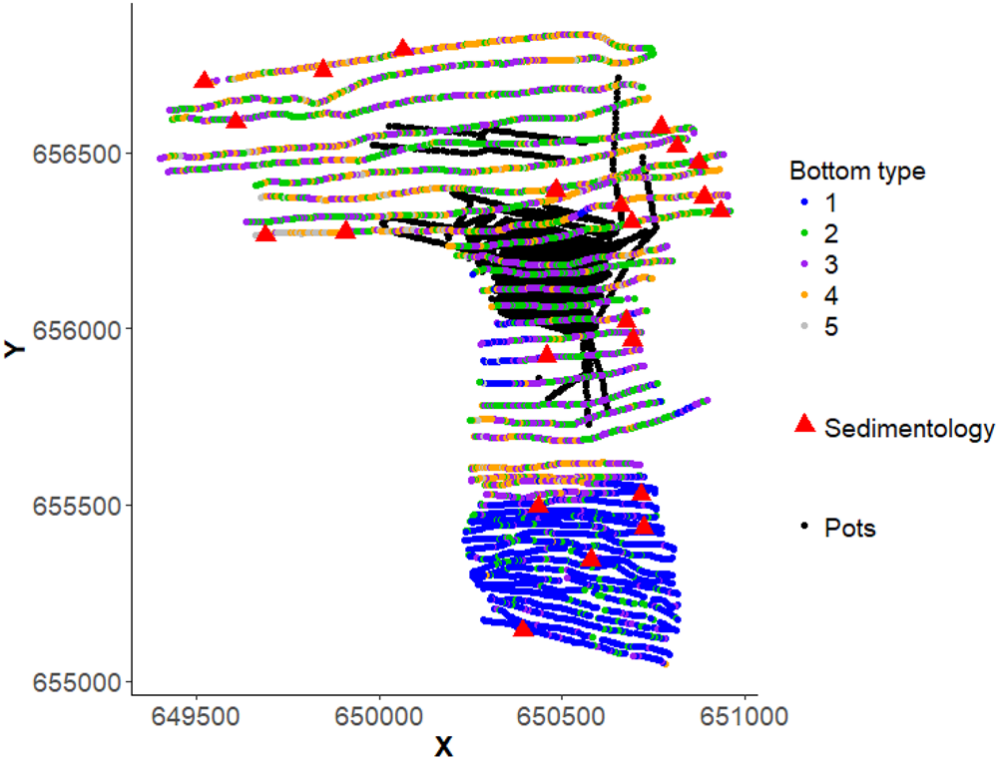
Habitat mapping of both fished area containing *N. norvegicus* and surrounding area with little or no *N. norvegicus*. Acoustic methods with unsupervised classification into 5 bottom classes are mapped with the coloured dots. The fished area is defined by black dots (one dot per pot fished). Red triangles indicate 22 ‘sedimentology’ stations where grabs and sediment size analysis was used to ground-truth acoustic mapping. Latitude/longitude co-ordinates are given as easting (X) and northing (Y) representing easting (X) and northing (Y) according to the Irish Transverse Mercator projection. Both acoustic bottom classes and sedimentology datasets are available at figshare^21^.

Acoustic data were collected using the Biosonics echosounder on the 21st and 22nd of July 2014 at depths varying from 2.8–23.1 m across the greater Study Area (Fig. 2). A battery was used onboard to provide a consistent power supply to allow collection of acoustic data with minimal noise and interference. The transducer was mounted on a pole attached to the transom of a 6 m rib and towed at a running depth of approximately 1 m at a speed of 4-5 knots. Navigation data was supplied by a Garmin echosounder using DGPS with an update rate of 1 second. Parallel tracks were run from west to east along the study site with a typical depth between 8 and 16 m. Every effort was made to keep the engine revolutions per minute (RPM) constant since the engine noise can influence the echogram. An unsupervised categorisation of 5 classes was chosen to represent a good balance between likely sediment classes and overfitting the noise in the data.

### Sedimentology

Sediments analysis was compliant with the North East Atlantic Marine Biological Analytical Quality Control scheme (NMBAQCS). Ground-truthing (i.e. sediment size categorisation) of acoustic data was carried out in August 2014 at 22 stations (Fig. 2). At each station, sediment was sampled with a single grab sample (0.1 m^2^ Van Veen grab). Visual inspection confirmed the presence of larger particulates in the sediment samples which means that samples were split and analysed with a combination of laser particle sizing (LPS) by means of laser diffraction, as well as by and wet and dry sieving (WDS). All samples were homogenised before analysis. Samples were halved and the first portion was analysed by the WDS method of ^26^. This involved oven drying the entire portion of the sediment at 100 °C for 24 h and weighing to get a dry weight. This was followed by addition of 10 ml of 1% solution of sodium hexametaphosphate (NaPO_3_)_6_, which aids dispersion of the clay particles. The sediment suspension was then stirred for 10 minutes using a mechanical stirrer before being left to stand overnight. On the following day the mixture was stirred again for 10 min and then sieved through a 500 micron (μm) mesh sieve to remove the fine fraction. The coarser fraction remaining on the mesh was rinsing thoroughly with fresh water to remove the dispersion agent. This sediment was dried again for another 24 hours at 100 °C. The sediment distributions coarser than 500 μm of the oven dried samples were determined by dry sieving. A collecting pan and a series of Phi Wentworth sieves (ranging from 4 mm to 500 μm) were stacked and loaded with dried material, with each sample left in an automated mechanical shaker for 10 minutes. Following shaking, all contents were brushed out of sieves and weighed separately. The fraction of sediment finer than 500 µm was determined by subtraction.

The particle size distribution of the sediment <500 µm was determined using the LPS method of ^27^. For each sediment sample, three replicate aliquots of the <500 μm fraction were analysed using a Malvern Mastersizer 2000 with a Hydro-G dispersion unit (Malvern Instruments Ltd, UK.). Sediment was added to the dispersion unit until obscuration reached between 15% and 18%. For each aliquot, the measurement cycle was 3 × 30,000 scans. The <500 µm LPS data for the replicate aliquots were averaged and converted to whole phi size class equivalents (500 to 250 µm, 250 to 125 µm, 125 to 63 µm, <63 µm) using GRADISTAT v8.0© statistical package^28^. The two data sets were then merged. The LPS data was normalised to the <500 μm WDS sieve percentage and then combined with the > 500 μm sieve fractions. The merged LPS and WDS distribution data were expressed as percentage weights within full Phi classes ranging between 4 and −2 Phi units (<63 μm to >4 mm)^28^. These data were processed using GRADISTAT and software output provided the percentage of the sediment in standard sediment size categories such as sand, silt, clay and gravel using conventional Folk sediment classes^28,29^ as well as sediment size statistics such as mean size, degree of homogeneity, and degree of sorting *via* skew and kurtosis of the size distribution.

## Data Records

Detailed explanation of dataset “***N. norvegicus*** **sampling**”^21^ is given in Table 1.

**Table 1:** Data on the size, growth, distance moved and catch information for *N. norvegicus*

Column	Data description	Typology of data
1	ID	Unique identifier for each pot fished
2	Longitude	Coordinate X (Irish Transverse Mercator projection; translated by a fixed offset)
3	Latitude	Coordinate Y (Irish Transverse Mercator projection; translated by a fixed offset)
4	Day	The fishing day on which a particular pot was fished (number)
5	String	The number string which was fished on a given day (number)
6	Pot	The number of pot on a given string (number)
7	Sex	Whether the individual was male or female (M/F). ‘U’ denotes individuals where sex was not recorded. Cells with ‘9999’ indicate that there was no catch on that Day/String/Pot
8	Carapace length	The measurement of the carapace from eye socket to rear end of the cephalic shield (mm). U indicates there was a catch but Carapace Length was not recorded. NA indicates no data available as pot had no catch
9	Notching performed	Whether or not v-notching was performed (Y/N)
10	Notch present	Whether or not v-notch was present (Y/N)
11	Berried	Whether or not a female was bearing embryos on the abdomen (Y/N)
12	Recently moulted	Whether or not the individual shows signs of recent moulting (Y/N)
13	Date captured	The date on which a tagged individual was recaptured after undergoing a period of growth in the wild (date DD/MM/YYYY)
14	Tagged recapture	Whether or not an individual was tagged with an internal tag (Y/N)
15	Days at liberty	The number of days a tagged individual was growing in the wild (number of days). U indicates a tagged individual with unknown days at liberty. NA indicates an individual lacking a tag therefore this parameter was not measured and is ‘not applicable’
16	Starting carapace length	The size of a tagged individual prior to release into the wild measured as carapace length (mm). NA indicates ‘not applicable’
17	Growth	The growth of a tagged individual while in the wild (=column 8-16) measured as carapace length (mm). ‘U’ indicates unknown as tag was lost. NA indicates ‘not applicable’.
18	Distance from release point	The distance between the mid-point at release and the point of recapture of a tagged individual (m). NA indicates ‘not applicable’

Detailed information of the first dataset related growth and distance moved of tagged individuals, positions of all pots bearing tagged, untagged, v-notched individuals as well as zero catch information (to enable catch-per-unit-effort or density calculations). This dataset enables growth to be linked with density in *N. norvegicus*.

The first dataset (***N. norvegicus*** **sampling**^21^) consists of:

1. 3289 females of mean size 37.60 ± 5.48 mm CL, 1446 males of mean size 40.87 ± 5.48 and 4 *N. norvegicus* individuals which were measured but sex was not recorded (denoted by ‘U’), with all the above captured during both 2014 and 2015. ‘Sex’ column records ‘9999’ for pots without any catch. Spatial positions for a total of 3300 (pots with no catch were denoted by ‘9999’).
2. 108 tagged females which were captured in 2014 of mean growth 1.34 ± 3.08 mm CL after an average of 340 days at liberty. 24 tagged females which were recaptured in 2015 of mean growth 4.36 ± 3.62 mm CL after an average of 724 days at liberty.
3. 96 tagged males which were captured in 2014 of mean growth 5.03 ± 3.09 mm CL after an average of 343 days at liberty. 12 tagged males which were recaptured in 2015 of mean growth 11.83 ± 3.78 mm CL after an average of 726 days at liberty.
4. Mean distance moved away from the release point of 231.47 ± 82.97 m by female tagged individuals.
5. Mean distance moved away from the release point of 232.41 ± 83.15 m by male tagged individuals.
6. 531 recaptures of v-notched individuals over 12 successive sampling dates.
7. ID refers to a unique identifier for each pot that was fished with information about the number of the fishing day, followed by the number of the string which contained pots, followed by the number of the pot; hence 1.1.1 means the first fishing day, the first string and the first pot on that string. (In this case 2 individual *N. norvegicus* were captured within the 1.1.1 pot).
8. Given that the population was sampled 14 times in 2014, and many individuals were neither tagged nor v-notched, unmarked individuals could be present more than once in the dataset.
9. Latitude/longitude co-ordinates are given as easting (X) and northing (Y) representing easting (X) and northing (Y) according to the Irish Transverse Mercator projection.
10. F means female, M means male, N means no/none, NA means no data available, and U means unknown.
11. When a pot had no catch, NA was recorded for Carapace Length.

Detailed explanation of dataset “**Acoustic data**”^21^ is given in Table 2.

**Table 2:** Data on the sea bottom classification by acoustic survey.

Column	Data description	Typology of data
1	Longitude	Coordinate X (Irish Transverse Mercator projection; translated by a fixed offset)
2	Latitude	Coordinate Y (Irish Transverse Mercator projection; translated by a fixed offset)
3	Date	Date on which sample was acquired (DD-MM-YYYY)
4	Time	Time at which sample was acquired (hr:min:sec)
5	Bottom type	Unsupervised bottom classification based on Biosonic acoustic survey

Detailed information of the second dataset related the bottom type according to Biosonics acoustic mapping.

The second dataset “**Acoustic data**”^21^ consists of:

1. 4894 bottom type classifications divided in a) 1360 type ‘1’, b) 1218 type ‘2’, c) 1365 type ‘3’, d) 829 type ‘4’, e) 122 type ‘5’.
2. Time stamped spatial positions for bottom types in X, Y coordinates, as before (see *N. norvegicus* sampling).

Detailed explanation of dataset “**Sedimentology**”^21^ is given in Table 3.

**Table 3:** Data on the sedimentology of the *N. norvegicus* study area.

Column	Data description	Typology of data
1	ID	The number of the station
2	Longitude	Coordinate X (Irish Transverse Mercator projection; translated by a fixed offset)
3	Latitude	Coordinate Y (Irish Transverse Mercator projection; translated by a fixed offset)
4	Folk & Ward Method mean	Mean sediment particle size (µm)
5	Folk & Ward Method sorting	Sediment sorting (standard deviation; µm)
6	Folk & Ward Method skewness	Sediment skewness
7	Folk & Ward Method kurtosis	Sediment kurtosis
8	Folk & Ward Method mean description	Mean sediment particle size (text)
9	Folk & Ward Method sorting description	Sediment sorting (text)
10	Folk & Ward Method skewness description	Sediment skewness (text)
11	Folk & Ward Method kurtosis description	Sediment kurtosis (text)
12	Percent gravel	The percentage of gravel in the sediment (%)
13	Percent sand	The percentage of sand in the sediment (%)
14	Percent mud	The percentage of mud in the sediment (%)
15-30	Sediment typology	The percentage of finer gradations of columns 12–14 (%)

Detailed information of the third dataset related sediment size and sorting information adjacent to the *N. norvegicus* fishing grounds, to enable ground-truthing of acoustic bottom types.

The third dataset “**Sedimentology**”^21^ consists of:

1. Mean sediment sizes for 22 grab samples which ranged from ‘very coarse silt’ (37.92–58.07 μm) to ‘coarse sand’ (554.4–799.9 μm) according to Folk and Ward categories^29^, along with sediment sorting, skew and kurtosis, described in both numeric and text typologies.

2. Percentage of the sediment in each grab sample which fell into a graded series of mud, sand and gravel classes.
3. Spatial positions for grabs in X, Y coordinates, as before (see *N. norvegicus* sampling).

## Technical Validation

Fishing was always carried out in conditions ‘suitable’ for fishing (in less than Beaufort Scale 4). To quality control tagging procedures, all individuals were scanned immediately after tagging to ensure the tag was indeed present and any individuals which did not have a tag present, for some reason, were re-tagged or rejected. Tagged individuals were held for several days afterwards, to ensure that they were in good condition and that they bore the tagging procedure well, before being scanned once again prior to controlled release into the wild. In order to investigate any negative effects of the tags, particularly on growth, an extra 232 individuals were held for 12 months at an aquaculture facility. Tags caused no negative effects on growth or mortality compared with a control group^30^. Also, scanning checks for tag retention in a captive tagged population^30^ were made approximately bi-monthly. These checks indicated that, once initial retention was good, tags were rarely if ever lost thereafter. During the acoustic classification of bottom types, the number of bottom types was chosen to correspond to the number of categories for which ground-truthed samples were available, i.e. 5.

## Usage Notes

Data are provided in CSV format. Care should be taken with conversion of columns containing DD/MM/YYYY data from csv files. Precise coordinates of the studied *N. norvegicus* are not shown to protect anonymity of the fishing grounds; however the spatial offset used for all latitude/longitude co-ordinates is the same to allow datasets to be overlain spatially. All spatial coordinates are given as X, Y using the Irish Transverse Mercator projection. A conversion to other projections can be done using Geographic Information System software such as QGIS. Lincoln-Petersen mark-recapture methods may also be used for density calculations within the fishing grounds^22,23^. CPUE may be estimated from data via the ratio of (re)captured *N. norvegicus* density and pot density, both resulting from a 2-D kernel density estimator. Farmer^31^ provides a conversion between Carapace Length and Total Body Length.

## ISA-Tab metadata file

Download metadata file
